# Local treatment with a polycarbophil-based cream in postmenopausal women with genitourinary syndrome of menopause

**DOI:** 10.1007/s00192-020-04282-9

**Published:** 2020-03-23

**Authors:** Tipatai Yodplob, Kun Sirisopana, Mutita Jongwannasiri, Pokket Sirisreetreerux, Wit Viseshsindh, Wachira Kochakarn

**Affiliations:** Division of Urology, Department of Surgery, Faculty of Medicine, Ramathibodi Hospital, Mahidol University, Bangkok, Thailand

**Keywords:** Genitourinary syndrome of menopause, Vaginal moisturizer, Vaginal lubricant, Polycarbophil-based cream, Vaginal dryness, Vulvovaginal atrophy

## Abstract

**Introduction and hypothesis:**

Genitourinary syndrome of menopause (GSM) is a common problem associated with lower urinary tract and gynecological symptoms due to the decrease in estrogen production in postmenopausal women. Topical estrogen therapy is shown to improve these symptoms; nonetheless, there are limited data on the efficacy of nonhormonal moisturizers in these patients.

**Methods:**

A prospective cohort study was conducted to compare the symptoms of GSM before and after treatment with a polycarbophil-based cream in 42 women. The quality of life (QoL) and sexual scores were obtained from the Thai version of the International Consultation on Incontinence Modular Questionnaire-Lower Urinary Tract Symptoms (ICIQ-LUTS) along with uroflow measurements before and 4 and 12 weeks after treatment.

**Results:**

Significant improvements in ICIQ-LUTSqol scores were observed after 4 weeks (9.38 ± 7.47 vs 6.76 ± 5.77; *p* = 0.017) and 12 weeks (10.03 ± 7.49 vs 5.97 ± 4.02; *p* = 0.002) when compared with the baseline values before treatment. The ICIQ-LUTS sexual scores were also improved after treatment at 4 weeks (2.29 ± 2.26 vs 0.88 ± 1.34; *p* < 0.001) and 12 weeks (2.13 ± 2.22 vs 0.42 ± 0.81; *p* < 0.001) compared with the baseline scores. No differences in ICIQ-LUTSqol and sexual scores were observed between the 4- and 12-week treatment groups.

**Conclusion:**

The polycarbophil-based cream improved the overall LUTS and sexual symptoms in the patients with GSM, thus indicating that the nonhormonal polycarbophil-based cream may prove effective for the treatment for women with this condition.

## Introduction

Genitourinary syndrome of menopause (GSM) is a common problem in postmenopausal women with a prevalence of about 50% [1–3]. The pathophysiology of this syndrome is due to the decrease in estrogen production from both ovaries in postmenopausal women. Estrogen depletion affects several organs, including the vaginal and urethral mucosae, leading to a reduction in vaginal secretion, thinning of the vaginal and urethral mucosae, and vaginal dryness, inflammation, and obstruction. Patients with this syndrome usually develop lower urinary tract symptoms, including increased urinary urgency and frequency, nocturia, urinary incontinence, urinary tract infection, vaginal dryness and irritation, and dyspareunia [4]. These symptoms are bothersome and affect the patients’ quality of life (QoL) [5].

Genitourinary syndrome of menopause is clinically diagnosed by obtaining the clinical history of the patient and by conducting physical and pelvic examinations. The common pelvic examination findings in patients with GSM as a result of estrogen depletion are vaginal dryness, loss of vaginal rugae, increased vaginal pH, leukorrhea, urethral or vaginal atrophy, meatal stenosis, and urethral prolapse. Subject-reported symptoms and clinical findings were categorized into three groups, external genitalia symptoms (vaginal dryness, pruritus vulva, leukorrhea), sexual symptoms (loss of libido, dyspareunia, bleeding during intercourse), and urological symptoms (urgency, frequency, nocturia, urinary tract infection) [6].

The treatment of GSM can be classified into two types [3, 6]: hormone replacement therapy, which can be further divided into systemic and local preparations, and nonhormonal therapy, which includes lifestyle modifications and the use of steroids, laser therapy, or vaginal moisturizers [3, 7, 8]. Previous studies have revealed that local hormone replacement therapy significantly improves the symptoms of patients with GSM [9, 10].

A polycarbophil-based cream is a hormone-free vaginal moisturizer composed of purified water (78.82%), polycarbophil, carbopol, glycerol, mineral oil, hydrogenated palm oil, glycerides, and sorbic acid. Previous studies have demonstrated the safety of this cream in the absence of any cytotoxic effects [11–13]. However, studies on the efficacy of the polycarbophil-based cream in terms of improvements in urinary symptoms are limited. The primary objective of this study was to evaluate the efficacy of this cream for the treatment of GSM, and the secondary objective was to assess the changes in uroflowmetry after the treatment. Our hypothesis is that nonhormonal polycarbophil-based cream will improve the symptoms in this group of patients.

## Materials and methods

Our study is a prospective single-cohort study. Approval for the study was obtained from the Committee for Research at the Faculty of Medicine, Ramathibodi Hospital, Mahidol University. All inclusion criteria had to be met as follows: women with one or more external genitalia or sexual symptoms; those with one or more urinary symptoms; and those who agreed to participate in this study. Patients who were allergic to polycarbophil-based cream, could not undergo uroflowmetry, and had been treated with a topical estrogen cream or vaginal moisturizer within 3 months prior to this study were excluded.

Forty-four women diagnosed with GSM between July 2017 and October 2018 were asked to participate in this study. Two of them were excluded because they refused to participate in this study. All 42 women were treated with a topical vaginal polycarbophil-based moisturizing cream. The patients were instructed to apply the topical vaginal moisturizer to the vagina and urethral opening once every 3 days before bedtime.

**Fig. 1:**
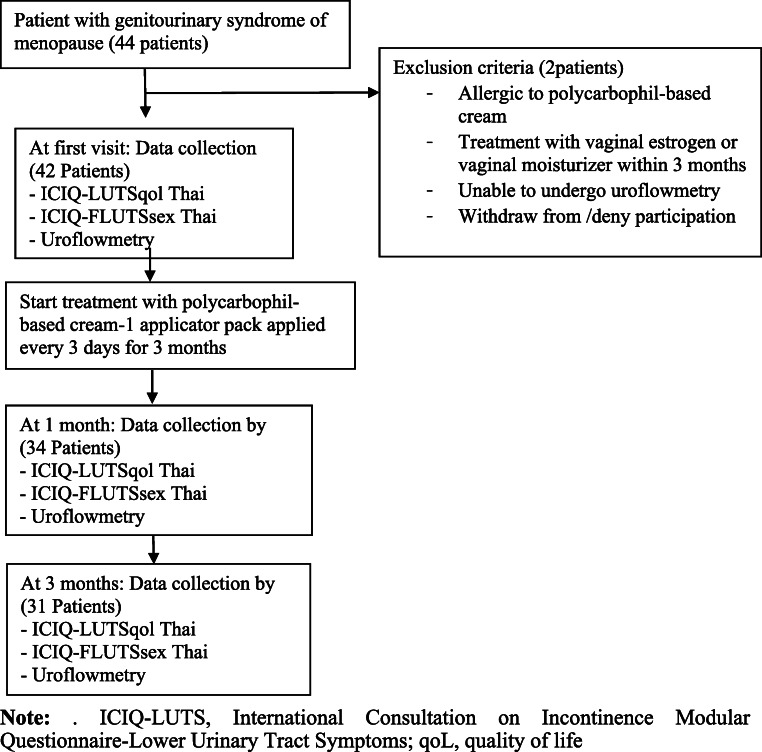
Flow chart of the study design.* ICIQ-LUTS* International Consultation on Incontinence Modular Questionnaire-Lower Urinary Tract Symptoms,* ICIQ-FLUTS* International Consultation on Incontinence Questionnaire Female Lower Urinary Tract Symptoms Modules, * qoL* quality of life

At the beginning of the study, data comprising the clinical characteristics of the patients, such as age, weight, height, body mass index (BMI), last menstrual period, and clinical symptoms of GSM (external genitalia symptoms, sexual symptoms, and urological symptoms), were collected. Baseline QoL and sexual scores of the Thai version of the International Consultation on Incontinence Modular Questionnaire-Lower Urinary Tract Symptoms (ICIQ-LUTS) [14] were collected directly by a doctor to evaluate the pretreatment symptom score. These questionnaires are composed of ascaling score of 1–10 to evaluate each urological, QoL and sexual symptom with a total of 22 questions. Uroflowmetry (Aquarius TT Laborie®) was performed in all the patients to evaluate the maximum urine flow and the void, as well as PVR urine volume. Uroflowmetry was evaluated using spontaneous void volume. PVR was measured by bladder scan (PORTASCAN™3D). All patients were informed about the study follow-up protocol and were encouraged to co-operate with the schedule. A few days before follow-up, patients were called by phone to remind them about the appointment. After 4 and 12 weeks of treatment, the patients were instructed to visit the outpatient department to re-evaluate the ICIQ-LUTSqol score, ICIQ-LUTSsex score, and uroflowmetry results. A flow chart of the study design is presented in Fig. 1.

The characteristics of the patients with the continuous variables are expressed as mean ± standard deviation (SD), and the categorical variables are expressed as frequency (%). The scores of the ICIQ-LUTSqol, ICIQ-LUTSsex, and uroflowmetry before and after treatment (4 and 12 weeks) with the polycarbophil-based cream were compared using the paired *t* test. Statistical analysis was conducted using the STATA program version 14 (StataCorp, College Station, TX, USA). The significance value (*p* value) was set at <0.05.

## Results

Baseline data were collected on 42 postmenopausal women (age, 66.08 ± 9.20 years; BMI, 24.05 ± 3.19 kg/m^2^; and duration of menopause, 17.68 ± 9.93 years from onset). Before treatment, the overall and bother baseline ICIQ-LUSqol scores were 9.38 ± 7.47 and 28.26 ± 24.15 respectively, whereas the corresponding ICIQ-LUTSsex scores were 2.29 ± 2.46 and 6.09 ± 6.92 respectively. The uroflowmetry values for void volume, maximum flow rate, and PVR urine volume were 261.59 ± 158.70 ml, 21.69 ± 10.74 ml/s, and 31.82 ± 41.01 ml respectively (Table 1).

**Table 1:** Baseline subject and clinical characteristics

Characteristic	Data
Demographics (*N* = 42)	
Age (years), mean ± SD	66.08 ± 9.20
Body weight (kg), mean ± SD	57.38 ± 8.37
Height (cm), mean ± SD	154.41 ± 5.86
BMI (kg/m^2^), mean ± SD	24.05 ± 3.19
Menstruation period (years), mean ± SD	17.68 ± 9.93
External genitalia, *n* (%)	
Dryness	38 (90.48)
Irritation/burning	17 (40.48)
Tenderness	4 (9.52)
Pruritic vulvae	3 (7.14)
Leukorrhea	6 (14.29)
Erythema	1 (2.38)
Fusion of labia	1 (2.38)
Sexual, *n* (%)	
Loss of libido	2 (16.67)
Loss of arousal	1 (8.33)
Lack of lubrication	5 (41.67)
Dyspareunia	6 (50.00)
Dysorgasmia	1 (8.33)
Pelvic pain	1 (8.33)
Urological, *n* (%)	
Frequency	22 (52.38)
Urgency	16 (38.10)
Nocturia	36 (85.71)
Dysuria	8 (19.05)
Hematuria	1 (2.38)
SUI/UUI	22 (52.38)
Post-void dribbling	3 (7.14)
Recurrent	1 (2.38)

BMI body mass index, SUI stress urinary incontinence, UUI urge urinary incontinence

After 4 weeks of treatment with the polycarbophil-based cream, the mean ± SD overall ICIQ-LUTSqol score was significantly improved from a baseline (*n* = 34) of 9.38 ± 7.47 to 6.76 ± 5.77 (*p* = 0.017), and the bother score was significantly improved from 28.26 ± 24.15 to 20.18 ± 21.33 (*p* = 0.025; Table 2). Likewise, the mean overall and bother ICIQ-LUTSsex scores were significantly improved, from 2.29 ± 2.46 to 0.88 ± 1.34 (*p* < 0.001) and from 6.09 ± 6.92 to 2.12 ± 3.46 (*p* < 0.001) respectively. As presented in Table 2, no significant changes in void volume and maximum flow were observed, whereas the PVR urine was significantly increased (*p* = 0.028) after use of the cream.

**Table 2:** Comparison of mean International Consultation on Incontinence Modular Questionnaire (ICIQ) scores and uroflowmetry parameters before and after (4 weeks and 12 weeks) treatment with the polycarbophil-based cream

	Before treatment (*n* = 34)	4th week (*n* = 34)	*p* value	12th week (*n* = 31)	*p* value
ICIQ-LUTSqol Thai (mean ± SD)					
Overall score	9.38 ± 7.47	6.76 ± 5.77	0.017*	5.97 ± 4.02	0.002*
Bother score	28.26 ± 24.15	20.18 ± 21.33	0.025*	18.77 ± 17.19	0.004*
ICIQ-LUTSsex Thai (mean ± SD)					
Overall score	2.29 ± 2.46	0.88 ± 1.34	<0.001*	0.42 ± 0.81	<0.001*
Bother score	6.09 ± 6.92	2.12 ± 3.46	<0.001*	1.06 ± 1.75	<0.001*
Uroflowmetry (mean ± SD)					
Void volume (ml)	261.59 ± 158.70	296.78 ± 139.39	0.232	298.87 ± 148.11	0.038*
Qmax (ml/s)	21.69 ± 10.74	23.12 ± 9.91	0.087	22.73 ± 10.47	0.093
PVR (ml)	31.82 ± 41.01	52.15 ± 60.66	0.028*	36.97 ± 43.88	0.378

Boldfaced *p* value denotes statistical significance (*p* < 0.05) at both the 4th week and the 12th week compared with baseline

*Qmax* maximum urine flow rate,* PVR* post-void residual urine Volume,* SD* standard deviation

*Comparison between groups using the paired* t* test

After 12 weeks of treatment with the cream, the mean overall and bother ICIQ-LUTSqol scores were significantly improved from a baseline (*n* = 34) of 10.03 ± 7.49 to 5.97 ± 4.02 (*p* = 0.002) and from 30.61 ± 23.99 to 18.77 ± 17.19 (*p* = 0.004) respectively. Significant improvements in the mean overall ICIQ-LUTSsex score from 2.13 ± 2.22 to 0.42 ± 0.81 (*p* < 0.001) and the mean bother score from 5.65 ± 6.36 to 1.06 ± 1.75 (*p* < 0.001) were observed. Furthermore, no significant changes in PVR urine maximum flow rate were noted; alternatively, the void volume was significantly increased from 246.93 ± 156.06 to 298.87 ± 148.11 (*p* = 0.038; Table 2).

No significant differences in scores were observed between 4 and 12 weeks of treatment, except for the PVR urine, which was significantly decreased from 53.74 ± 63.37 to 36.97 ± 43.88 (*p* = 0.022; Table 3).

**Table 3:** Comparison of mean International Consultation on Incontinence Modular Questionnaire (ICIQ) scores and uroflowmetry parameters between 4 and 12 weeks of treatment

	4th week (*n* = 31)	12th week (*n* = 31)	*p* value
ICIQ-LUTSqol Thai (mean ± SD)			
Overall score	7.06 ± 5.89	5.97 ± 4.02	0.236
Bother score	21.48 ± 21.84	18.77 ± 17.19	0.310
ICIQ-FLUTSsex Thai (mean ± SD)			
Overall score	0.68 ± 1.14	0.42 ± 0.81	0.174
Bother score	1.74 ± 3.23	1.06 ± 1.75	0.112
Uroflowmetry (mean ± SD)			
Void volume (ml)	291.23 ± 128.65	298.87 ± 148.11	0.715
Qmax (ml/s)	22.84 ± 9.82	22.73 ± 10.47	0.899
PVR (ml)	53.74 ± 63.37	36.97 ± 43.88	0.022*

Boldfaced *p* value denotes statistical significance (*p* < 0.05)

*Qmax* maximum urine flow rate,* PVR* post-void residual urine volume,* SD* standard deviation

*Paired* t* test

## Discussion

The main objectives of localized GSM treatment to alleviate the genital symptoms and to restore the vaginal environment to its normal healthy condition. Although vaginal estrogen therapy is effective for the treatment of GSM [10, 15, 16]. In patients with estrogen-sensitive malignancy, use of the nonhormone-based moisturizers and lubricants is considered first-line therapy, whereas estrogenized therapies are reserved for those women whose symptoms are refractory to first-line treatment [17–24]. Vaginal moisturizers rehydrate the dry mucosal tissue; they are absorbed into the skin and adhere to the vaginal lining, thereby mimicking natural vaginal secretions by changing the fluid content of the endothelium and lowering the vaginal pH [9].

In a previous study, Origoni et al. [25] demonstrated significant improvements in the symptoms of patients with GSM, both objectively (using the vaginal health index) and subjectively (using the visual analog scale), after 2 months of treatment with a hyaluronic acid-based vaginal lubricant. In addition, a significant improvement in the QoL of these patients was reported with a patient satisfaction score of 95% at the end of their study. The findings of the current study are in agreement with the results of the aforementioned study; significant improvements in both QoL and sexual symptoms were observed after 4 weeks of treatment with the vaginal moisturizer in the present study. These improvements were also observed 12 weeks after the treatment.

The void volume was significantly increased after 12 weeks of treatment. However, the maximum urine flow rate was not significantly altered after 4 and 12 weeks of treatment. Although PVR urine was significantly increased after 4 weeks of treatment when compared with the baseline values, these changes might not be clinically significant.

A strength of this study is that its prospective design. Also, the study uses validated questionnaires to evaluate patients’ symptoms as well as good patient compliance and follow-up. A limitation of the study was that comparisons between the use of a placebo and standard treatment (hormonal vaginal preparation) were not made in this study, as it might have been difficult to interpret the true efficacy of the treatment method and lack of control group of women without treatment in this study. The study was focus on voiding and sexual symptoms but less on gynecological symptoms. Additional well-designed randomized controlled studies are warranted.

## Conclusion

Use of nonhormonal vagina polycarbophil-based moisturizing cream for the treatment of genitourinary symptoms of menopause resulted in significant improvements in validated lower urinary tract and sexual quality of life measures. This study suggests that a nonhormonal vaginal polycarbophil-based moisturizing cream might be effective for the treatment of patients with GSM.
